# Transient loss of consciousness in boxer dogs with hypothyroidism: 3 cases (2015–2018)

**DOI:** 10.1186/s12917-025-04931-5

**Published:** 2025-12-15

**Authors:** Harriet A. O. Broome, Hannah Hodgkiss-Geere, Yolanda Martinez Pereira, Erin O’Connell

**Affiliations:** 1 Small Animal Teaching Hospital, University of Liverpool, Leahurst Campus, Chester High Road, Neston, CH64 7TE UK; 2 Hospital for Small Animals, The Royal Dick School of Veterinary Studies, Easter Bush Campus, Midlothian, EH25 9RG USA

**Keywords:** Syncope, Levothyroxine, Collapse, Thyroxine, Thyroid stimulating hormone

## Abstract

**Background:**

Three Boxers, two neutered females and one neutered male, aged from seven to eleven years, were presented for investigation of transient loss of consciousness.

**Case presentation:**

In each case, physical examination revealed no significant abnormalities and echocardiography, and electrocardiography did not identify a cause of transient loss of consciousness. Twenty-four-hour electrocardiogram (Holter) monitoring was performed in all three dogs. One dog had an episode of transient loss of consciousness whilst wearing the Holter, during which second-degree atrioventricular block and sinus bradycardia were observed. Based on clinicopathological testing, all three dogs were diagnosed with hypothyroidism. Once hypothyroidism was diagnosed all dogs were started on levothyroxine. In all cases the transient loss of consciousness resolved or improved once the treatment was started.

**Conclusion:**

Hypothyroidism should be considered as a differential diagnosis in Boxers presenting with transient loss of consciousness. Treating hypothyroid Boxers may decrease or resolve the clinical signs.

## Background

Syncope, a common condition in Boxers, is described as a brief loss of consciousness due to decreased cerebral blood flow [1]. Transient loss of consciousness (TLOC) is a more general term used to describe all disorders resulting in loss of consciousness, regardless of the underlying cause [2, 3]. Diagnosis of TLOC and syncope can be challenging due to the many aetiologies and intermittent nature. Accordingly, a diagnosis is only reached in 40 to 50 per cent of dogs presenting with syncope [4]. Transient loss of consciousness is especially common in Boxers, in part due to the high prevalence of increased vagal tone leading to vasovagal syncope, subaortic stenosis (SAS) and arrhythmogenic right ventricular cardiomyopathy (ARVC) in the breed [5, 6]. Boxers presenting for syncope, collapse and exercise intolerance without a diagnosis are significantly more likely to suffer from premature death compared with other breeds, making an accurate diagnosis imperative [7].

There are many known causes of TLOC, including neurological conditions like epilepsy, cardiorespiratory disease, for example dilated cardiomyopathy, and metabolic diseases like hypoglycemia. Whilst hypothyroidism is known to cause cardiogenic changes [8, 9] it is not a commonly recognised cause of TLOC in dogs. Hypothyroidism is the result of impaired production of the thyroid hormones, triiodothyronine (T_3_) and thyroxine (T_4_). Due to the diffuse and diverse role of the thyroid hormones in homeostasis, hypothyroidism is characterised by a variety of clinical signs however, it most frequently presents with a combination of metabolic and dermatological changes [10, 11]. A spectrum of other conditions have also been attributed to hypothyroidism including anaemia, megaesophagus, peripheral neuropathy and keratoconjunctivitis sicca [10, 12, 13, 14].

Tests routinely used to achieve a diagnosis of hypothyroidism include measurement of total thyroxine (tT_4_), free thyroxine (fT_4_) and thyroid stimulating hormone (TSH) [15, 16, 17]. However, interpretation of test results can be challenging due to several factors that affect the concentration of thyroid hormones, including nonthyroidal illness, administration of certain drugs, and age [18] and can lead to misdiagnosis [19]. The challenges involved in diagnosis make understanding the presentation and associated conditions important.

The objective of this case series was to describe Boxer dogs that presented for syncope which resolved or improved following the diagnosis and treatment of hypothyroidism.

## Case one

### Case presentation

A 27 kg (BCS 4/9) 10-year-old female neutered Boxer was examined following one episode of TLOC after rest. There were no significant findings on physical examination.

### Clinicopathologic analysis

Complete blood count and serum biochemistry were performed at admission (Table 1) using a commercial veterinary laboratory (IDEXX Laboratories, Kornwestheim, Germany). Analyses were conducted with an automated haematology analyser (Siemens ADVIA 2120i, Siemens Healthcare Diagnostics, Erlangen, Germany). The dog had mild hypercholesterolemia, a moderate increase in lipase, mild hyperphosphataemia and a borderline decrease in total calcium. Cardiac troponin I concentration was measured using a commercial veterinary laboratory (IDEXX Laboratories, Kornwestheim, Germany) and an automated immunoassay analyser (Immulite 2000 XPi, Siemens Healthcare Diagnostics, Erlangen, Germany). The value was elevated but not considered consistent with myocarditis or acute myocardial injury [20].

**Table 1:** A summary of the abnormalities found during routine serum biochemistry, complete blood count (CBC) and cardiac troponin I results

	Hct	RBC	HGB	Reticulocyte count	Serum potassium	Serum cholesterol	Serum ALP	Serum urea	Serum Lipase	Serum phosphorus	Serum Creatinine	Total calcium	Cardiac Troponin I
**Case one**	0.50 L/L (RI:0.35–0.55)	6.81 × 10^12^/L (RI:5.4-8.0)	15.3 g/dL (RI:12–18)	0.02 × 10^12^/L (RI: 11–110)	4.87mmol.L (RI:3.6–5.6)	6.3mmol/L (RI:3.2–6.2)	50.0U/L (RI:<=130)	5.0mmol/L (RI:3.1–10.1)	463.0U/L (RI:0.1–200)	1.74mmol/L (RI:0.8–1.6)	79µmol/L (RI: 44–133)	2.3mmol/L (RI:2.31–2.84)	0.18ng/mL (RI:<0.15)
**Case two**	0.29 L/L (RI:0.35–0.55),	3.91 × 10^12^/L (RI:5.4-8.0)	9.7 g/dL (RI:12.18)	0.01 × 10^9^/L (RI: 11–110)	3.7mmol/L (RI:3.8–5.3).	4.0mmol/L (RI:3.2–6.5)	102U/L (RI:0-100)	2.9mmol/L (RI:3.5-6)	96U/L (RI:0-100)	0.92mmol/L (RI:0.8-2.0)	67µmol/L (RI:44–159)	2.58mmol/L (RI:2.2–2.7)	0.688ng/L (RI:0.01–0.15)
**Case three**	0.44 L/L (RI:0.35–0.55)	6.89 × 10^12^/L (RI:5.4-8.0)	15.1 g/dL (RI:12–18)	100.6 × 10^9^/L (RI: 11–110)	3.8mmol/L (RI:3.5–5.8)	10.66mmol/L (RI:2.8–8.2)	647 U/L (RI:23–212)	4.9mmol/L (RI:2.5–9.6)	1411U/L (RI:200–1800)	1.3mmol/L (RI:0.8–2.2)	122 µmol/L (44–159)	3.07 mm/L (RI:1.98-3.00)	Not performed

ALP = Alkaline phosphatase. HGB = heamoglobin. RBC = Red blood cell. Hct = Heamatocrit

### Diagnosis of hypothyroidism

Canine TSH concentration was borderline increased and total T4 (tT4) was low-normal, as measured using an automated immunoassay analyser (Immulite 2000 XPi, Siemens Healthcare Diagnostics, Erlangen, Germany). Free T4 (fT4) was subsequently assessed by equilibrium dialysis, and repeat measurements of tT4, fT4, and TSH were performed one month later by a commercial veterinary laboratory (Axiom Veterinary Laboratories, Newton Abbot, UK). On the second occasion, tT_4_ was decreased and TSH was within normal limits. Free T_4_ was decreased on both occasions (Table 2).

**Table 2:** A summary of the serum tT_4,_ TSH and fT_4_ results

	tT_4_	TSH	Repeated tT_4_	Repeated TSH	fT_4_
**Case one**	7.2nmol/L (RI:5.0–44.0)	0.51ng/mL (RI:0.03–0.5)	8.4nmol/L (RI:10.0–55.0)	0.26ng/mL (RI:0.01–0.6)	5.2pmol/L (RI:6.6–40)
**Case two**	5.25nmol/L (RI:6.5–44.0)	2.11ng/mL (RI:0.03–0.50)	Not performed	Not performed	Not performed
**Case three**	6.72nmol/L (RI:5–44.0)	1.86ng/mL (RI:0.03–0.5)	Not performed	Not performed	Not performed

fT4 = Free T_4_. TSH = Thyroid stimulating hormone. tT_4_ = Total T_4_. The reference ranges are specific to the laboratory used to perform the test

### Diagnostic imaging findings

Doppler echocardiography (GE Vivid E95, GE Healthcare, Chicago, Il, USA) (Table 3), thoracic radiography and abdominal ultrasound were performed and revealed no significant abnormalities.

**Table 3:** Selected measurements from the echocardiography performed on the three cases

	LA/Ao	LVSd (mm)	LVIDs (mm)	LVIDd (mm)	%FS (%)	LVD_ind	IVSd_ind	LVESV_ind (ml/m^2^)	TAPSE (cm)
**Reference range**	1.05–1.75^(Rishniw *et al.* 2019)^	12.1 (8.3–16.1)^(Cunningham *et al.* 2008)^	24.5 (16.7–33.0)^(Cunningham *et al.* 2008)^	39.6+/-3.4 ^(Smets *et al.* 2014)^	31.3+/- 4.6 ^(Smets *et al.* 2014)^	1.27–1.85^(Cunningham *et al.* 2008)^	0.29–0.59^(Cunningham *et al.* 2008)^	36+/-7^**(Smets*et al.*2014)**^	RR for 27 kg: 1.5 (1.2–2.6). RR for 46 kg: 1.8 (1.2–2.7)^(Feldhutter *et al.* 2022)^
**Case one**	1.21	9.1	31.0	43.3	28	1.64	0.41	34.5	1.74
**Case two**	1.19	14.8	23.4	38.0	39	1.27	0.60	27.0	N/A
**Case three**	1.52	10.44	27.1	39.9	32	1.29	0.41	14.9	2.00

LA/Ao = Left atrium/aorta. LVSd = Left ventricular septum diastole. LVIDs = Left ventricular internal diameter end systole. LVIDd = Left ventricular internal diameter end diastole. FS = Fractional shortening. LVD_ind = Left ventricular diameter indexed to body weight. IVSd_ind = Interventricular septum end diastole indexed to body weight. LVESV_ind = left ventricular end systolic volume indexed to body weight. TAPSE = Tricsupid anular plane systolic excursion. RR = Reference range

### ECG

Sinus rhythm with a mean heart rate of 79 bpm and occasional ventricular premature complexes (VPCs).

### Holter ECG (24-hour ECG) findings

A 24-hour ECG was performed prior to diagnosis and treatment of hypothyroidism. The predominant rhythms were sinus rhythm and sinus arrhythmia with ventricular ectopy (112/24 hours; RI ≤ 91/24 hours) that occurred singly (similar to ECG findings) [21]. No episodes of syncope occurred whilst wearing the Holter (Table 4).

**Table 4:** Selected 24-hour ECG results from the first 24-hour ECG performed in each case

	Predominant rhythm	Minimum heart rate (bpm)	Maximum heart rate (bpm)	Mean heart rate (bpm)	VPCs	Number of sinus pauses > 3s
**Case one**	Sinus arrhythmia	47	158	79	Aberrant total: 145 VPCs: 112	0
**Case two**	Sinus arrythmia	54	179	87	Aberrant total: 20 Single VPCs: 11 Two couplets 1 ventricular run	3
**Case three**	Sinus and sinus arrythmia	61	193	91	Aberrant total: 62 VPCs: 23	0

VPCs = Ventricular premature complexes

### Treatment and outcome

Following the diagnosis of hypothyroidism, treatment with 0.5 mg (0.02 mg/kg) levothyroxine (Leventa, MSD Animal Health, Milton Keynes, UK) PO every 24 h was started. After starting treatment no further episodes of TLOC occurred and blood tests performed two months after diagnosis confirmed appropriate supplementation with suppression of thyroid stimulating hormone (TSH) (< 0.03ng/ml, RI: <0.50) and normal free thyroxine (fT4) (36.8pmol/L, RI: 7.7–47.6). At this point, a further 24-hour ECG was performed, with similar results to the first.

### Follow up

Several months after diagnosis, case one acutely deteriorated after being found dyspneic and unable to walk. Unfortunately, a diagnosis was not reached, and the dog was euthanased.

## Case two

### Case presentation

A 41 kg (BCS 7/9) 7-year-old female neutered Boxer presented with a two-week history of episodes of TLOC; the episodes occurred when standing after being asleep and lasted for around two minutes. The owner had reported that she was quieter in herself. There was a II/VI left basilar systolic murmur on cardiopulmonary auscultation.

### Clinicopathologic analysis

Complete blood count and serum biochemistry were performed at admission (Table 1) using an automated haematology analyser (Advia 2120i, Siemens Healthcare Diagnostics, Erlangen, Germany) and analysed by a commercial laboratory (IDEXX Laboratories, Kornwestheim, Germany). Findings included a mild, non-regenerative anaemia and a moderate increase in alkaline phosphatase activity (ALP). Cardiac troponin I concentration (Table 1) was increased, but not consistent with myocarditis or acute myocardial injury, as measured using an automated immunoassay analyser (Immulite 2000 XPi, Siemens Healthcare Diagnostics, Erlangen, Germany) [20].

### Diagnosis of hypothyroidism

Total T_4_ was less than the reference interval and thyroid stimulating hormone was increased (Canine total T4, Immulite 2000 XPi, Siemens Healthcare Diagnostics, Erlangen, Germany) (Table 2).

### Diagnostic imaging findings

Mild aortic stenosis was identified on Doppler echocardiography (Table 3) (GE Vivid E95, GE Healthcare, Chicago, Il, USA). Abdominal ultrasound and thoracic radiography were performed and revealed no significant abnormalities.

### ECG

Sinus rhythm with a mean heart rate of 93 bpm and no VPCs.

### Holter ECG (24-hour ECG) findings

The predominant rhythm was sinus rhythm. The dog had two syncopal events whilst wearing the Holter. During these events the ECG showed periods of sinus slowing and high grade second degree atrioventricular block (AVB) with idiojunctional and idioventricular escape mechanisms. During the first episode the escape rate was 25 bpm but returned to sinus rhythm after six beats. Upon returning to sinus rhythm, the rate was initially bradycardic (46 bpm) but then increased to 85 bpm. The total episode occurred over a period of 30 s. During the second syncopal episode the escape rate was 40 bpm. It then returned to sinus arrythmia (66 bpm), and this recovery happened in 15 s. The longest R to R interval was 4.2 s (Table 4).

### Treatment and outcome

Subsequent to the 24-hour ECG the dog was started on 5 mg of terbutaline (Bricanyl; AstraZeneca, Cambridge, UK) PO every 12 hours due to the suspicion of a neurocardiogenic component. For the week following commencement of treatment, there was an initial period without TLOC however, following this an episode of weakness was noted and the dose of terbutaline was increased to 5 mg PO every 8 h. A follow up 24-hour ECG was performed which recorded 13 ventricular premature complexes, 1 couplet, and 1 supraventricular premature complex over the full recording. No episodes of TLOC occurred during the ECG recording. The owner reported progressive lethargy, and a worsening anemia was documented (HCT 0.204 L/L (0.35–0.55), RBC 2.52 × 10^12^/L (5.4-8.0), Hgb 6.3 g/dL [12, 13, 14, 15, 16, 17, 18], MCV 81.1fL (65–75), MCHC 33.4 g/dL (32–37), reticulocyte count 68 × 10^9^/L (11–110)) in addition to hyperkeratosis of the nasal planum. At this time the dog was diagnosed with hypothyroidism and therapy with 0.8 mg (0.02 mg/kg) of levothyroxine PO every 12 hours (Thyforon, Dechra, Shrewsbury, UK) was initiated. A significant improvement in the dog’s demeanor and energy levels were noted two weeks after starting treatment with levothyroxine and no further syncopal episodes were recorded. An improvement in the anaemia was documented (HCT 0.236 L/L (0.35–0.55, was 0.204), RBC 2.82 × 10^12^/L (5.4-8.0, was 2.52), Hgb 7.3 g/dL (12–18, was 6.3), MCV 83.8fL (65–75), MCHC 30.7 g/dL (32–37), reticulocyte count 84.6 × 10^9^/L (11–110)). Three months after this appointment the haematology showed a normal haematocrit (0.407 L/L, Ref. 0.38–0.57) and the owner reported that she had continued to be more playful. The terbutaline dose was tapered over six weeks and then stopped. No further episodes of TLOC or weakness were reported.

### Follow up

Case two was presented as an emergency six months after starting treatment with levothyroxine (two months after the last presentation) and was diagnosed with high-grade abdominal lymphoma and received palliative treatment. She was euthanised 9 days later.

## Case three

### Case presentation

A 46 kg (BCS 9/9) 11-year-old male Boxer was evaluated after experiencing four episodes of TLOC over a 7-month period. All episodes started with coughing and lasted for around 30 s before rapid, complete recovery. There was a II/VI left basilar systolic murmur on cardiopulmonary auscultation.

### Clinicopathologic analysis

Complete blood count and serum biochemistry were performed at admission (Table 1) using an automated haematology analyser (Advia 2120i, Siemens Healthcare Diagnostics, Erlangen, Germany), with analysis conducted by a commercial laboratory (IDEXX Laboratories, Kornwestheim, Germany). Findings included mild hypercholesterolaemia, mild hypokalaemia, and a mild decrease in urea.

### Diagnosis of hypothyroidism

Total T_4_ was low normal and thyroid stimulating hormone was increased (Canine total T4, Canine total T4, Immulite 2000 XPi, Siemens Healthcare Diagnostics, Erlangen, Germany) (Table 2).

### Diagnostic imaging findings

Degenerative mitral valve disease (ACVIM stage B1) was identified on Doppler echocardiography (GE Vivid E95, GE Healthcare, Chicago, Il, USA) (Table 3) [22]. Computed tomography (CT) of the thorax and abdomen was performed and revealed a small left thyroid/parathyroid nodule but no further abnormalities.

### ECG

Sinus rhythm with a mean heart rate of 180 bpm and the presence of frequent VPCs of right ventricular origin.

### Holter ECG (24-hour ECG) findings

The 24-hour ECG was performed two weeks after treatment for hypothyroidism had commenced but euthyroidism had not been achieved. The trace from the 24-hour Holter ECG showed that the predominant rhythms were sinus and sinus arrhythmia. There were 62 ventricular complexes of which 23 were VPCs (RI ≤ 91/24 hours) [21], one was a triplet (instantaneous heart rate of 219 bpm), and the remaining complexes were due to ventricular bigeminy (Table 4).

### Treatment and outcome

The diagnosis of hypothyroidism was made following presentation for investigation of TLOC. Treatment with 0.8 mg (0.02 mg/kg) of levothyroxine (Thyforon, Dechra, Shrewsbury, UK) PO every 24 h was started. Ten days later the dog was presented following two further episodes of TLOC. These differed from the previous episodes in that they were not preceded by coughing and there was a period of disorientation post-TLOC. Total T_4_ was repeated and was increased (83.3nmol/L, RI: 5–44), with TSH in the upper end of the reference range (0.388ng/mL, RI: 0.03–0.50). The dose of levothyroxine was adjusted to 0.4 mg PO every 12 h (no decrease in total dose). A 24-hour ECG was performed at this time (105 VPCs, mostly singular monomorphic, two couplets). Two weeks later tT_4_ was high-normal and the hypothyroidism was considered well controlled (tT_4_ 48.8nmol/L, RI: 13–51; TSH 0.33ng/mL, RI: <0.50).

### Follow up

A 24-hour ECG was repeated six months later following one episode of TLOC, and the results indicated likely ARVC (999 aberrant complexes, 229 VPCs with near R on T, 218 complexes were less premature, 5 couplets with an instantaneous rate of 264 bpm, 1 triplet with an instantaneous rate of 348 bpm at the time of stress and excitement). Seven instances of bradycardia (Heart rate < 45 bpm for > or equal to 4 beats) were also noted. Treatment was not started as it was not considered indicated at this time.

## Discussion

This case series describes three Boxer dogs that were presented for investigation of TLOC. All three dogs were diagnosed with hypothyroidism and TLOC appeared to improve or they had no further TLOC following treatment with levothyroxine. However, a direct causal relationship cannot be definitively established.

Whilst TLOC is common in Boxer dogs, an association with hypothyroidism has not previously been reported. Thyroid hormones have direct and indirect effects on the cardiovascular system [8, 9], and hypothyroidism can lead to cardiogenic abnormalities, including bradycardia and atrioventricular block (AVB) in both dogs and humans [23, 24, 25, 26]. Aetiologically, it is hypothesised that reduced beta-adrenergic stimulation in hypothyroid states allows parasympathetic tone to dominate, prolonging the refractory period and predisposing to AVB [27]. In human medicine, this heightened parasympathetic activity is well documented [28, 29], and a similar pattern has been observed in dogs. One case report described a hypothyroid Dalmatian exhibiting bradycardia, pause events, AVB, and ventricular ectopy, all of which resolved following levothyroxine treatment as sympathetic tone increased, and parasympathetic tone diminished [26].

Comparable findings were seen in case two of our series, in which 24-hour ECG during syncopal episodes revealed high-grade second-degree AVB and sinus slowing. The longest R–R interval was 4.2 s, insufficient alone to cause TLOC, suggesting the combination of AVB and sinus slowing as the likely cause, consistent with type 1B syncope, characterised by advanced AVB and progressive sinus rate reduction [3].

In the other two cases no episodes of TLOC were reported whilst the Holter was in place, so it is not possible to ascertain the cause of TLOC in these Boxers. In case one and two there was resolution of TLOC after treatment with levothyroxine. However, as case one only had one episode prior to diagnosis with hypothyroidism, it cannot be certain that the treatment resulted in resolution of TLOC.

In this case series, the only breed investigated were Boxers. As a brachycephalic breed, they are identified as having increased vagal tone [5]. It is possible that the combination of the two disorders results in further increase in parasympathetic activity, the consequence of which is a decreased threshold for episodes of TLOC. Furthermore, brachycephalic breeds have several anatomical abnormalities such as nasal stenosis and elongated soft palates, which can result in hypoxia [30]. This could be a contributing factor the TLOC [31].

Anemia occurs in 32 to 44 per cent of dogs with hypothyroidism [12] and was seen at initial presentation in case two. Severe anemia can cause TLOC [32] in dogs but is unlikely to be a significant contributing factor in this case.

Arrhythmogenic right ventricular cardiomyopathy is an inherited myocardial abnormality common in Boxers, characterised by ventricular arrhythmias which can result in TLOC [33]. Neurocardiogenic bradycardia is another common cause of TLOC in Boxers which occurs when there is reflex vasodilation without a relative increase in heart rate [34]. In case three the TLOC improved following treatment with the correct dose of levothyroxine. The first two Holter readings did not indicate the presence of ARVC and although there was variation between the two Holters, up to 80 per cent variation between recordings has been reported in Boxers with this disease [35]. However, six months later one further syncopal episode was reported after initiation of the correct dose of levothyroxine and the final Holter recording revealed likely ARVC and periods of bradycardia, either of which could result in TLOC. It is feasible that this dog had hypothyroidism, and then later developed ARVC and neurocardiogenic bradycardia concurrently, which would have contributed to the final TLOC episode reported.

It is not yet clear what would cause TLOC in hypothyroid Boxers, but it is likely that cardiovascular changes such as an increase in parasympathetic tone play a role. The presence of an already increased vagal tone in the breed combined with the occurrence of other abnormalities common to Boxers, such as ARVC and neurocardiogenic bradycardia, could further increase the likelihood of TLOC compared with other dogs with hypothyroidism. Further research is required to investigate if Boxers with hypothyroidism have higher vagal tone compared with other breeds with the same condition.

There are several limitations to this case series. The diagnosis of hypothyroidism in case one is more equivocal than in the other two cases. However, it should be noted that that reference ranges for the tT4 are lower than most other laboratories which confounds results that would be considered consistent with hypothyroidism elsewhere. This applies to case one and three, where a tT4 of 7.2nmol/L and 6.7nmol/L, respectively, would generally be considered low but are within the reference range for the laboratory used. Furthermore, it is not possible to say with certainty that hypothyroidism is the cause of the episodes of TLOC. For example, case one only had one episode of TLOC before the diagnosis of hypothyroidism. Additionally, two of the dogs did not have episodes of TLOC whilst wearing the Holter, meaning the reference standard for diagnosis of TLOC was non-diagnostic [30]. Finally, due to the retrospective nature there were some aspects of the history that were not reported, which would have been useful in understanding the cases.

When evaluating a Boxer with syncopal episodes, it is important to assess thyroid function. While correcting hypothyroidism in such cases may lead to a reduction in the frequency or severity of syncopal episodes, further research is needed to establish a definitive link. It is also important to continue to try to identify any concurrent diseases that may contribute to the syncopal episodes.

## Data Availability

Data is provided within the manuscript.

## Abbreviations

ALP: Alkaline phosphatase
ARVC: Arrhythmogenic right ventricular cardiomyopathy
AVB: Atrioventricular block
CBC: Complete blood count
CT: Computed tomography
fT_4_: Free thyroxine
IMHA: Immune meditated hemolytic anemia
SAS: Subaortic stenosis
RI: Reference interval
TSH: Thyroid stimulating hormone
T_4_: Thyroxine
tT_4_: Total thyroxine
TLOC: Transient loss of consciousness
T_3_: Triiodothyronine
VPCs: Ventricular premature complexes

## References

[1] Wray J. Differential diagnosis of collapse in the dog: cardiovascular and miscellaneous causes. Pract. 2005;27(3):128–35.

[2] Bassetti CL. Transient loss of consciousness and syncope. Handb Clin Neurol. 2014;119:169–91. 24365296 10.1016/B978-0-7020-4086-3.00013-8

[3] Perego M, Porteiro Vázquez DM, Ramera L, Lombardo SF, Pane C, Bontempi LV, Santilli RA. Heart rhythm characterisation during unexplained transient loss of consciousness in dogs. Vet J. 2020;263:105523. 32928492 10.1016/j.tvjl.2020.105523

[4] Martin MWS, Corcoran BM. *Notes on cardiorespiratory diseases of the dog and cat.* 2nd rev ed. Blackwell Publishing; 2005.

[5] Doxey S, Boswood A. Differences between breeds of dog in a measure of heart rate variability. Vet Rec. 2004;154(23):713–7. 15214514 10.1136/vr.154.23.713

[6] Bussadori C, Quintavalla C, Capelli A. Prevalence of congenital heart disease in boxers in Italy. J Vet Cardiol. 2001;3(2):7–11. 19081337 10.1016/S1760-2734(06)70014-5

[7] Barnett L, Martin MW, Todd J, Smith S, Cobb M. A retrospective study of 153 cases of undiagnosed collapse, syncope or exercise intolerance: outcomes. J Small Anim Pract. 2011;52(1):26–31. 21175622 10.1111/j.1748-5827.2010.01013.x

[8] Karlapudi SK, Srikala D, Rao DST. Hypothyroidism—a cause for cardiomyopathy in dogs: fouryear study. Vet World. 2012;5:742–7.

[9] Monge Utrilla O, Romaniega Maeso I, López Segura MB, Martín Otero JL. Canine dilated cardiomyopathy secondary to hypothyroidism: review of three clinical cases. J Cardiol Cardiovasc Res. 2022;3(3):1–8.

[10] Panciera DL. Hypothyroidism in dogs: 66 cases (1987–1992). J Am Vet Med Assoc. 1994;204(5):761–7. 8175472

[11] Dixon RM. Epidemiological, clinical, haematological and biochemical characteristics of canine hypothyroidism. Vet Rec. 1999;481:–. (verify journal, volume, pagination). 10.1136/vr.145.17.481 10596870

[12] Mooney CT. Canine hypothyroidism: a review of aetiology and diagnosis. N Z Vet J. 2011;59(3):105–14. 21541883 10.1080/00480169.2011.563729

[13] Jaggy A, Oliver JE, Ferguson DC, Mahaffey EA, Glaus T Jr. Neurological manifestations of hypothyroidism: a retrospective study of 29 dogs. J Vet Intern Med. 1994;8(5):328–36. 7837108 10.1111/j.1939-1676.1994.tb03245.x

[14] Huber E, Armbrust W, Forster JL, Ribiere T, Grosclaude P. Resolution of megaesophagus after treatment of concurrent hypothyroidism in a dog. Schweiz Arch Tierheilkd. 2001;143(10):512–4. 11680912

[15] Dixon RM, Mooney CT. Evaluation of serum free thyroxine and Thyrotropin concentrations in the diagnosis of canine hypothyroidism. J Small Anim Pract. 1999;40(2):72–8. 10088086 10.1111/j.1748-5827.1999.tb03040.x

[16] Nelson RW, Ihle SL, Feldman EC, Bottoms GD. Serum free thyroxine concentration in healthy dogs, dogs with hypothyroidism, and euthyroid dogs with concurrent illness. J Am Vet Med Assoc. 1991;198(8):1401–7. 1648049

[17] Peterson ME, Melián C, Nichols R. Measurement of serum total thyroxine, triiodothyronine, free thyroxine, and Thyrotropin for diagnosis of hypothyroidism in dogs. J Am Vet Med Assoc. 1997;211(11):1396–402. 9394888

[18] Díaz Espineira MM, Mol JA, Peeters ME, Pollak YW, Iversen L, van Dijk JE, et al. Assessment of thyroid function in dogs with low plasma thyroxine concentration. J Vet Intern Med. 2007;21(1):25–32. 17338146 10.1892/0891-6640(2007)21[25:aotfid]2.0.co;2

[19] Travail V, Fernandez Sanchez C, Costo JM, Valentine N, Conroy M, Lee V, et al. Assessment of the likelihood of hypothyroidism in dogs diagnosed with and treated for hypothyroidism at primary care practices: 102 cases (2016–2021). J Vet Intern Med. 2024;38(2):931–41. 38314891 10.1111/jvim.16993 PMC10937491

[20] Burgener IA, Kovacevic A, Mauldin GN, Lombard CW. Cardiac troponins as indicators of acute myocardial damage in dogs. J Vet Intern Med. 2006;20(2):277–83. 16594583 10.1892/0891-6640(2006)20[277:ctaioa]2.0.co;2

[21] Stern JA, Meurs KM, Spier AW, Koplitz SL, Baumwart RD. Ambulatory electrocardiographic evaluation of clinically normal adult boxers. J Am Vet Med Assoc. 2010;236(4):430–3. 20151866 10.2460/javma.236.4.430

[22] Atkins C, Bonagura J, Ettinger S, Fox P, Gordon S, Häggström J, et al. Guidelines for the diagnosis and treatment of canine chronic valvular heart disease. J Vet Intern Med. 2009;23(6):1142–50. 19780929 10.1111/j.1939-1676.2009.0392.x

[23] Schoenmakers N, de Graaff WE, Peters RH. Hypothyroidism as the cause of atrioventricular block in an elderly patient. Neth Heart J. 2008;16(2):57–9. 18335023 10.1007/BF03086119 PMC2245813

[24] Seol SH, Kim DI, Park BM, Kim DK, Song PS, Kim KH, et al. Complete atrioventricular block presenting with syncope caused by severe hypothyroidism. Cardiol Res. 2012;3(5):239–41. 28348695 10.4021/cr221w PMC5358139

[25] Stephan I, Nolte I, Hoppen HO. The effect of hypothyroidism on cardiac function in dogs. Dtsch Tierarztl Wochenschr. 2003;110(6):231–9. 12866255

[26] Panciera DL. An echocardiographic and electrocardiographic study of cardiovascular function in hypothyroid dogs. J Am Vet Med Assoc. 1994;205(7):996–1000. 7852177

[27] Correia Costa P, Waller SB, Rocha DG, Alves CC, Soares M, de Silva A, de Vasconcellos EG. AL. Dose of Levothyroxine restored autonomic balance in a dalmatian female dog with primary hypothyroidism. Acta Sci Vet 2020;48.

[28] Inukai T, Takanashi K, Kobayashi H, Fujiwara Y, Tayama K, Aso Y, et al. Power spectral analysis of variations in heart rate in patients with hyperthyroidism or hypothyroidism. Horm Metab Res. 1998;30(8):531–5. 9761386 10.1055/s-2007-978927

[29] Sahin I, Turan N, Kosar F, Taskapan C, Gunen H. Evaluation of autonomic activity in patients with subclinical hypothyroidism. J Endocrinol Invest. 2005;28(3):209–13. 15952403 10.1007/BF03345374

[30] De Melo Dias ML, Morris CFM, Moreti BM, de Espírito Santo AV, McManus C, de Almeida, et al. Anatomical, cardiovascular, and blood gas parameters in dogs with brachycephalic syndrome. Acta Sci Vet. 2016;44(4):1356.

[31] Moya A, Sutton R, Ammirati F, Blanc JJ, Brignole M, Dahm JB, Deharo JC, Gajek J, Gjesdal K, Krahn A, Massin M, Pepi M, Pezawas T, Ruiz Granell R, Sarasin F, Ungar A, van Dijk JG, Walma EP, Wieling W. Guidelines for the diagnosis and management of syncope. Eur Heart J. 2009;30(21):2631–7. 19713422 10.1093/eurheartj/ehp298 PMC3295536

[32] Assenmacher TD, Jutkowitz LA, Koenigshof AM, de Lucidi C, Scott MA. Clinical features of precursor-targeted immune-mediated anemia in dogs: 66 cases (2004–2013). J Am Vet Med Assoc. 2019;255(3):366–76. 31298643 10.2460/javma.255.3.366

[33] Basso C, Fox PR, Meurs KM, Towbin JA, Spier AW, Calabrese F, et al. Arrhythmogenic right ventricular cardiomyopathy causing sudden cardiac death in boxer dogs: a new animal model of human disease. Circulation. 2004;109(9):1180–5. 14993138 10.1161/01.CIR.0000118494.07530.65

[34] Thomason JD, Kraus MS, Surdyk KK, Fallaw T, Calvert CA. Bradycardia-associated syncope in 7 boxers with ventricular tachycardia (2002–2005). J Vet Intern Med. 2008;22(4):931–6. 18537877 10.1111/j.1939-1676.2008.0119.x

[35] Spier AW, Meurs KM. Evaluation of spontaneous variability in the frequency of ventricular arrhythmias in boxers with arrhythmogenic right ventricular cardiomyopathy. J Am Vet Med Assoc. 2004;224(4):538–41. 14989546 10.2460/javma.2004.224.538

